# Novel strategy to identify MHC class II-promiscuous CD4+ peptides from tumor antigens for utilization in vaccination

**DOI:** 10.1186/2051-1426-2-S3-P47

**Published:** 2014-11-06

**Authors:** Jashodeep Datta, Shuwen Xu, Julia H Terhune, Cinthia Rosemblit, Erik Berk, Elizabeth Fitzpatrick, Brian J Czerniecki

**Affiliations:** 1 University of Pennsylvania Perelman School of Medicine, PA, USA

## Background

Although cytotoxic CD8 T lymphocytes (CTL) were historically considered primary effectors of antitumor immunity, solely boosting CTL responses with CD8 vaccines in various tumor types has yielded unpredictable clinical results, possibly because CTLs function suboptimally without adequate CD4 T lymphocyte help. CD4 T-helper type 1 (Th1) cells secrete INF-γ/TNF-α, inducing tumor senescence and apoptosis. As such, successful incorporation of CD4 epitopes into cancer vaccine construction and generation of durable antigen-specific CD4 immunity remains a challenge. Using the extracellular domain (ECD) of HER3 as a candidate "oncodriver" tumor antigen, we sought to identify immunogenic HER3 peptides that demonstrate Class II promiscuity and generate anti-HER3 CD4 immunity for inclusion in vaccine development.

## Methods

A library comprising 123 overlapping 15 amino acid-long peptide fragments was generated from the HER3-ECD. Autologous monocyte-derived DCs from donors were matured to a type 1-polarized (DC1; IL-12 secreting) phenotype, and pulsed with HER3-ECD. Harvested DC1s were co-cultured with purified CD4 T cells. After 10 days, sensitized CD4 T cells were restimulated against immature DCs (iDC) pulsed with HER3 library peptide clusters or irrelevant CD4 peptide control. Th1 responses, measured by IFN-γ ELISA, were considered antigen-specific if IFN-γ production was at least twice that of irrelevant control.

## Results

**Figure 1:**
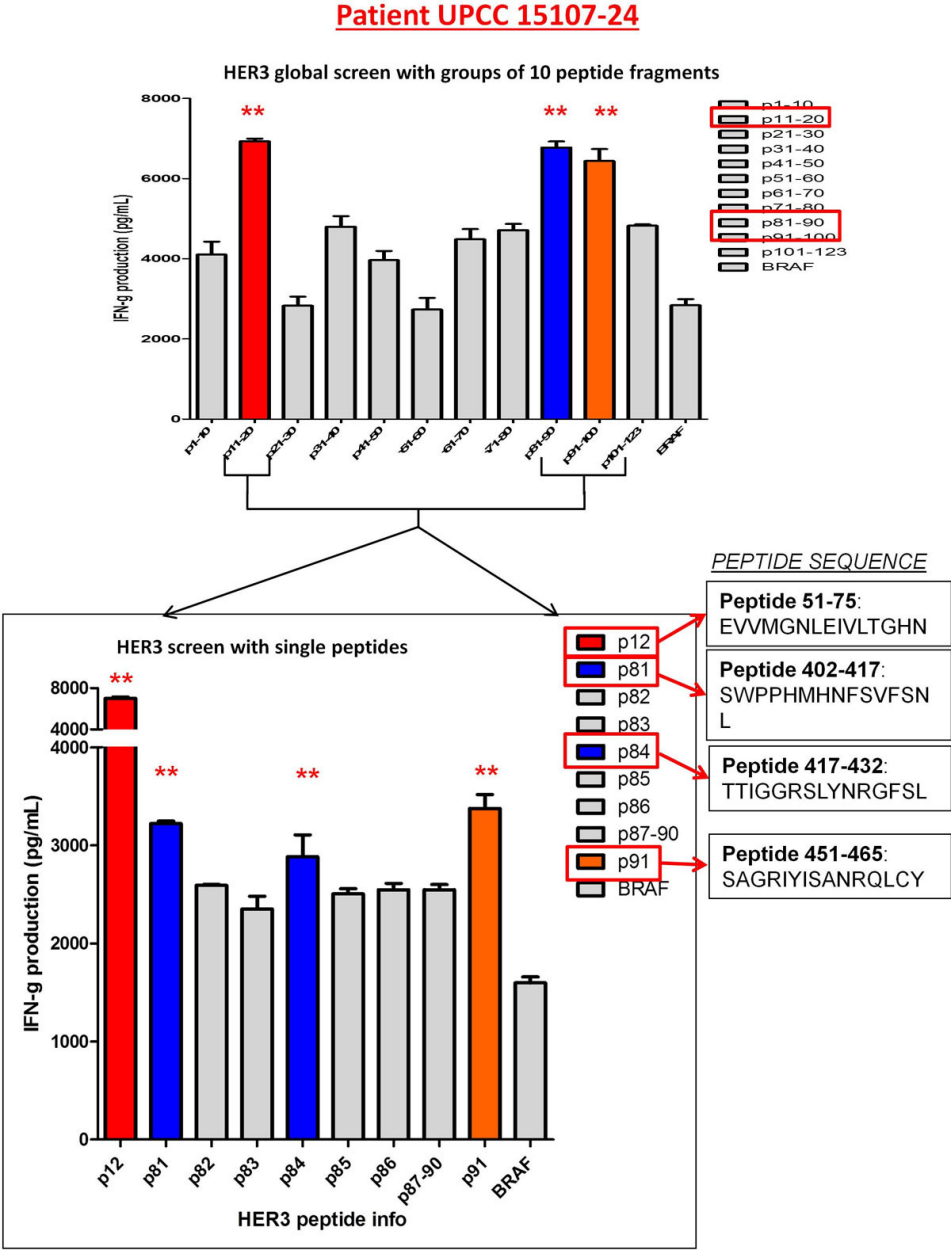
**Representative peptide screen**.

Th1 sensitization was initially performed in 5 breast cancer patients with known anti-HER3 *reactivity *in order to identify single immunogenic HER3 CD4 epitopes. To achieve this, HER3 ECD-specific CD4 Th1 were sequentially restimulated against 10-peptide clusters, narrowed to 3-peptide clusters, and ultimately to single immunogenic HER3 peptides. A representative peptide screen is depicted in Figure 1. Four immunogenic peptides - HER3(51-75), HER3(402-417), HER3(417-432), HER3(451-465) - were reproducibly identified and promiscuous across HLA-DR, DP, and DQ subtypes. When Th1 cells from 4 *non-HER3 reactive *donors were sensitized using DC1s pulsed with the four identified HER3 peptides, and subsequently challenged to recognize HER3 ECD-pulsed iDCs, all donors demonstrated successful sensitization not only to individual immunogenic HER3 peptides, but also recognized native HER3-ECD.

## Conclusions

DC1 pulsed with an overlapping tumor antigen-derived peptide library can identify promiscuous class II peptides for CD4 T cell vaccine development. In this study, immunogenic HER3 CD4 peptides effectively overcome immune tolerance to self-tumor antigens. Utilization of these HER3 CD4 peptides in vaccine construction warrants investigation in patients harboring HER3-overexpressing cancers. Additionally, these results represent a novel strategy to rapidly and reproducibly identify class II-promiscuous immunogenic CD4 epitopes from any tumor antigen for cancer immunotherapy using a DC1-Th1 platform.

